# Testing of Silicon Rubber/Montmorillonite Nanocomposite for Mechanical and Tribological Performance

**DOI:** 10.3390/nano11113050

**Published:** 2021-11-12

**Authors:** Avinash Shinde, I. Siva, Yashwant Munde, Vishal Deore, Mohamed Thariq Hameed Sultan, Ain Umaira Md Shah, Faizal Mustapha

**Affiliations:** 1 Center for Composite Materials, Kalasalingam Academy of Research and Education, Anand Nagar 6261286, TN, India; avinash.shinde@cumminscollege.in (A.S.); isiva@klu.ac.in (I.S.); 2 Department of Mechanical Engineering, Cummins College of Engineering for Women, Pune 411052, MH, India; Yashwant.Munde@cumminscollege.in (Y.M.); vishal.deore@cumminscollege.in (V.D.); 3 Department of Aerospace Engineering, Faculty of Engineering, Universiti Putra Malaysia, Serdang 43400 UPM, Selangor Darul Ehsan, Malaysia; ainumaira91@gmail.com (A.U.M.S.); faizalms@upm.edu.my (F.M.); 4 Laboratory of Biocomposite Technology, Institute of Tropical Forestry and Forest Products (INTROP), Universiti Putra Malaysia, Serdang 43400 UPM, Selangor Darul Ehsan, Malaysia; 5 Aerospace Malaysia Innovation Centre (944751-A), Prime Minister’s Department, MIGHT Partnership Hub, Jalan Impact, Cyberjaya 63000, Selangor Darul Ehsan, Malaysia

**Keywords:** silicon rubber, montmorillonite (MMT), sliding wear, mechanical properties, nanocomposites

## Abstract

Nanocomposite made by blending nano-montmorillonite (MMT) and Silicon Rubber (SR) for mechanical and tribological performance is explored in this work. Different configurations of MMT/SR nanocomposite, with 0, 0.5, 2 and 5 wt % of MMT are manufactured by two roll mixing methods. Noticeable improvement in the mechanical and tribological performance is observed, which is also justified by a morphological study of fractured and wear surfaces through SEM. Two percent of MMT loading is found to be the optimum content that shows excellent performance compared to other compositions. The performance improvement can be linked to the good interfacial interaction between the MMT and SR. Statistical modeling through ANOVA is carried out for tribological performance, which reveals the influence of load on the coefficient of friction (COF) and the influence of sliding distance on the wear rate.

## 1. Introduction

Synthetic elastomers like silicone rubber (SR) are commonly found in application in industries such as aerospace, medical, and electronics. Owing to their excellent environmental inertness, thermal stability, and insulating properties [1,2], most sectors prefer SR. Silicon rubber properties can be radically improved by adding nanofillers like carbon black (CB), carbon nanotube (CNT), graphite, nano C_e_O_2_ [3,4]. Similar works can be found in literature that show the influence of graphene oxide addition on resistivity and dielectric constant [5], thermal stability with the function of iron oxide and CNT filling [6], and tribological study [7] of various nanocomposites performed on the SR composite. All of these studies have revealed the usefulness of SR for different engineering applications.

The utility of SR for different applications can be improved by a deep analysis of its mechanical and tribological properties, among others. The mechanical and wear characteristic of rubber components can be one essential factor when deciding its candidature for a particular application. Adding graphene and nano-C_e_O_2_ as a filler in SR showed a decrease in the COF [4]. Reinforcement with CB enhanced the mechanical and tribological properties [8]. Graphite nanoplatelets improved the mechanical and abrasion resistance properties of the styrene-butadiene rubber [9]. The kinetic COF of the tubular rubber seal decreases with an increasing sliding velocity and reduces the loading level [10]. The wear properties of natural rubber and styrene-butadiene rubber were tested against different rocks as friction material and found to be improved [11]. The enhancement of mechanical, wear, and dielectric properties due to the addition of the exfoliated graphite in silicon rubber was investigated. The COF was observed to reduce with an increase in the wear rate and load. Simultaneously, the specific wear rate reduced, similar to COF, with graphite content but increased with an increase in load. The sliding velocity had a similar effect on both the COF and the wear rate, and it increased with an increase in the sliding velocity. The author correlated this improvement with good bonding between the matrix and the filler [12]. Ruben Sanchez-Hidalgo [13] demonstrated that if we can control the structure, the chemical composition, and the morphology of graphene material, the property of the SR can be tailored. Solution and flocculation processes were used for the preparation of the graphene silicon composite. The wear and mechanical characteristics depended on the reinforcement and agglomeration of the graphene [14]. The sonication process for processing polyacrylamide (PMA) reinforced with clay nanoparticles was used, and the effect of the sonication time and frequency was investigated. The rheological properties and nanoparticles dispersion were found to improve with an increase in the frequency [15].

Very few researchers have explored the capability of montmorillonite (MMT) for tribological application as a filler in polymers [16]. With increases in the concentration of MMT, the COF was found to decrease due to its lubricating behaviour. Sodium-montmorillonite (Na-MMT) nanoparticles were added to polyethersulfone (PES)/polytetrafluoroethylene (PTFE) to understand the thermal, mechanical, and tribological properties. All properties showed considerable improvement. The reduction in COF was observed with a rise in the normal load for Na-MMT/PES/PTFE composites. The author attributed this to the layered structure of the Na-MMT nanoparticles. A precisely opposite behaviour was shown by the Na-MMT/PES/PTFE composite. The authors observed that the response of both compositions to the sliding velocity was exactly contrary for the normal load. The weight, COF, and wear rate of the composite can be decreased with the addition of MMT in the matrix [16,17]. The effect of the grain size and distribution of Ag2s nanoparticles on the mechanical and tribological performance was studied. The improvement in the tribological performance was attributed to good mechanical properties and an ability to form a protective tribofilm [18]. Tribological properties of the graphene reinforced ceramic showed improvement because of the formation of a protective transfer layer, which acted as a lubricant between the contacting surfaces [19,20].

SR has wider suitability in aerospace, automotive, electrical, and electronic industries. There is no work reported on the effectiveness of MMT/silicon rubber nanocomposites for tribological applications to the best of the authors’ knowledge. The objective of this work is to investigate the mechanical and tribological behaviour of the silicon rubber–MMT nanocomposites. In this work, the weight percentage of MMT is set at 0.5, 2 and 5%. Pin-on-disc testing experiments are performed with different loading, sliding velocity, and sliding distance. The ANOVA experimental design technique is used for designing experiments and statistical modeling. Weight loss and friction force are calculated and used for the calculation of the specific wear rate. Manufacturing, testing, and results are discussed in detail in the following sections.

## 2. Materials and Methods

### 2.1. Material Details

SR with SH5060U grade was procured from Krupa Chemicals, Pune, India. Montmorillonite (MMT) was supplied by Ad-Nano Technologies Private Limited, Shimoga, India. The material properties as specified by the supplier in the datasheet are listed in Table 1. The curing agent used was Dicumyl Peroxide of 40% purity procured from Krupa Chemicals and used in the proportion as given in Table 2 with SR.

### 2.2. Preparation of Silicon Rubber–Nanoclay Composites

**Figure 1:**
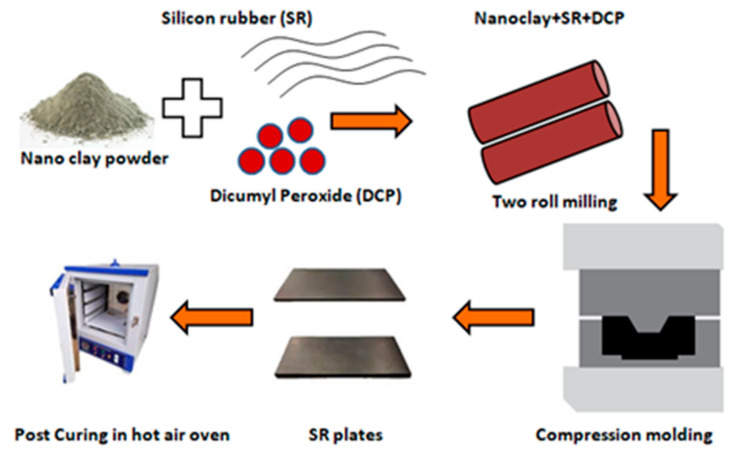
Schematic representation of SR–MMT manufacturing process.

The schematic representation of SR–MMT manufacturing is shown in Figure 1. SR with SH5060U grade and peroxide-based curing agent such as Dicumyl Peroxide (DCP) of 40% purity were compounded with different weight concentrations of nanoclay, viz., 0, 0.5, 2, 5% and are hereafter referred to as SR1, SR2, SR3, and SR4. The compounding formulation is listed in Table 2. The mixing was carried out for approximately 40 min on a two roll mill machine. The compound was cured in a SANTEC compression moulding machine (Santech Industries, Delhi, India) of 30 ton capacity. The temperature, pressure, and curing time were maintained at 170 degrees, 50 bar and 5 min, respectively; followed by the post-curing at 200 degrees for 4 h in a hot air oven (Make: Athena Technology, Model: ATAO-3 S/G).

### 2.3. Characterizations

The characteristics of MMT/SR focused on were physical (hardness), mechanical and tribological properties. To confirm the uniform dispersion of the nanofiller, morphology tests, viz., SEM and X-ray diffraction (XRD), were performed. The details of these techniques are summarized in the following paragraphs.

#### 2.3.1. Morphology Testing

XRD was carried out to analyze the maturity and formation of the nanocomposites. XRD analysis was performed in Bruker AXS D8 advance X-ray diffractometer (Bruker India Scientific Pvt Ltd, Mumbai, India) with X-ray source-Cu Kαradiation (λ = 0.154 nm). The scattering was plotted as a function of scattering angle 2θ.

#### 2.3.2. Mechanical Testing

**Figure 2:**
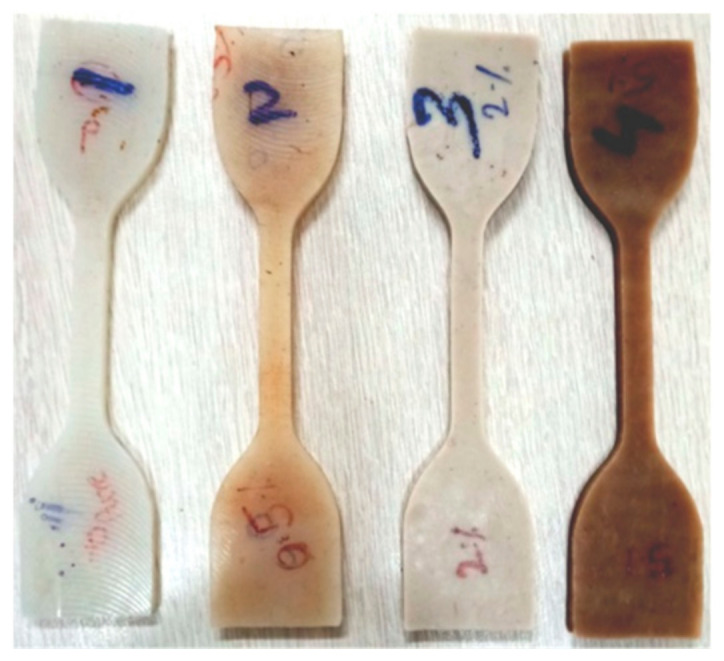
Samples prepared for tensile testing.

A sample (as shown in Figure 2) preparation and testing for tensile and tear test was performed according to ASTM D 412 and ASTM D624, respectively. The tensile test was performed at a speed of 450 mm/min. Shore hardness testing was completed according to ASTM D2240. All tests were carried out at a 450 mm/min strain rate with an environmental condition of 24 °C and 54% humidity.

#### 2.3.3. Experimental Design of Tribological Study

**Figure 3:**
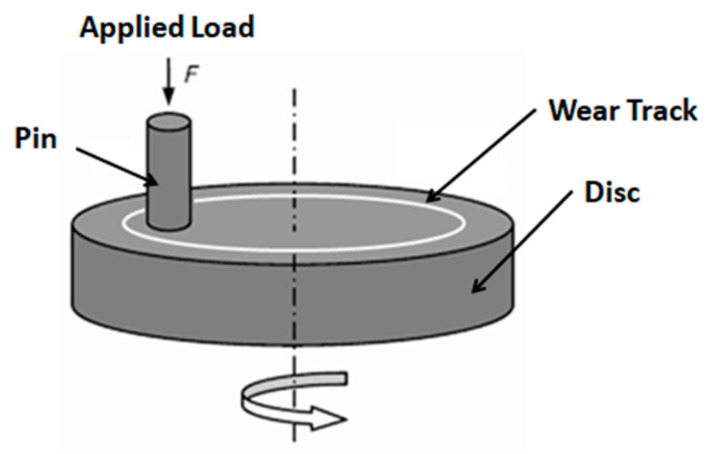
Schematic representation of SR–MMT manufacturing process.

The Schematic representation of pin-on-disc machine and its different components is shown in Figure 3. Levels of control parameters are listed in Table 3. Taguchi’s L_16_ orthogonal array was used to perform the design of tribology experiments, as shown in Table 4.

A pin-on-disc wear testing machine was used, and tests were performed according to the ASTM G99 standard. The samples were cut in the form of a rectangular pin of 10 × 10 × 30 mm. The rubbing disc was made of EN-31 of hardness 60 HRC. All tests were performed at room temperature with general conditions of temperature and humidity. Samples were weighed before and after the experimental runs. An analytical weighing balance was used for accurate measurement of weight loss. The friction force ($F_{f}$) recorded by the controller was used for the calculation of friction coefficient μ, as given in Equation (1) below

(1) $$\mu =\frac{F_{f}}{F_{n}}$$

where $F_{n}$ is the normal force applied to the specimen.

Also, specific wear rate (W) was calculated by using Equation (2):

(2) $$W=\frac{Weight\;Loss}{Density\times Normal\;Load\times Sliding\;Distance}$$

#### 2.3.4. SEM and EDS Analysis

The FESEM analysis was carried out on an ultra-high-resolution FEI Nova NanoSEM 450 machine (FEI, Hillsboro, OR, USA). The machine’s resolution is 1.0 nm at 15 kV, 1.4 nm at 1 kV, and 1.8 nm at 3 kV and 30 Pa. EDS spectra were obtained on the same machine with excellent energy resolution (123 eV at Mn Kα and 45 eV at C Kα), and element detection range from 4 Be to 95 Am.

## 3. Results and Discussion

### 3.1. X-ray Diffraction Studies

**Figure 4:**
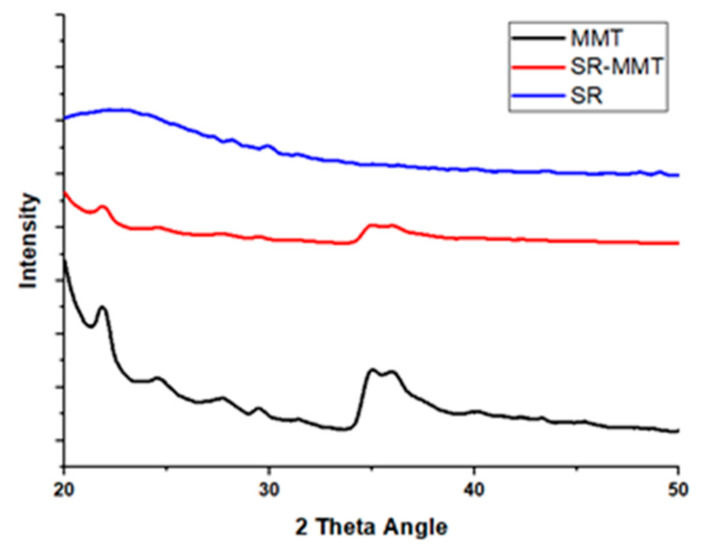
XRD pattern of pure SR, MMT powder and SR4 composition.

Figure 4 shows the XRD plot of intensity versus scattering angle for the pure SR, the SR4 composite, and MMT powder samples. MMT powder shows a high degree of crystallinity with a broad peak at 35° 2θ angles. A similar broad reflection is also observed in the SR4 composite, with somewhat lesser intensity, which implies individual MMT particles within the retained crystal structure. The peak corresponding to the SR is at around 30°, which is also observed in the SR4 composite. The existence of these individual constituent peaks in the SR4 composite confirms the correct formation of the composite.

### 3.2. Mechanical Properties

#### 3.2.1. Tensile Strength

**Figure 5:**
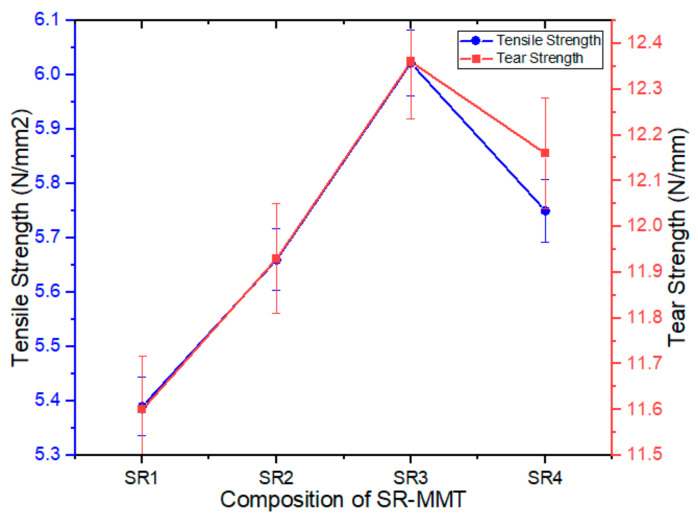
Variation of tensile strength and tear strength of SR–MMT nanocomposites with weight % of MMT.

The tensile strength of the SR–MMT nanocomposites is found to increase with an increase in the weight fraction of MMT (Figure 5). The tensile strength of SR1 was observed at 5.39 MPa, which increases to 5.66 and 6.02 MPa at SR2 and SR3 compositions, respectively, and then decreases to 5.75 MPa for SR4. The highest value of tensile strength is 6.02 MPa which is 11.68% higher than the SR1 composition. As seen from the XRD results, the reduced interlayer distance for SR3 composition is the reason behind the increased tensile strength. This reduced interlayer distance improves the interlayer bonding between the matrix and the filler.

#### 3.2.2. Tear Strength

The tearing strength is initially found to increase with an increasing amount of MMT. The highest tear strength value is 12.36 MPa for the SR3 composition, 6.55% higher than for the pure SR. With a further increase in the MMT percentage, the tearing strength starts to decline and reaches 12.15 MPa. A better uniform dispersion of the MMT could explain this in the matrix for the SR3 composite. Reduction in the tear strength for the SR4 composition is attributed to the agglomeration in composite with an increase in the wt % of the MMT.

#### 3.2.3. Peak Load, Braking Load, Elongation at Break

**Figure 6:**
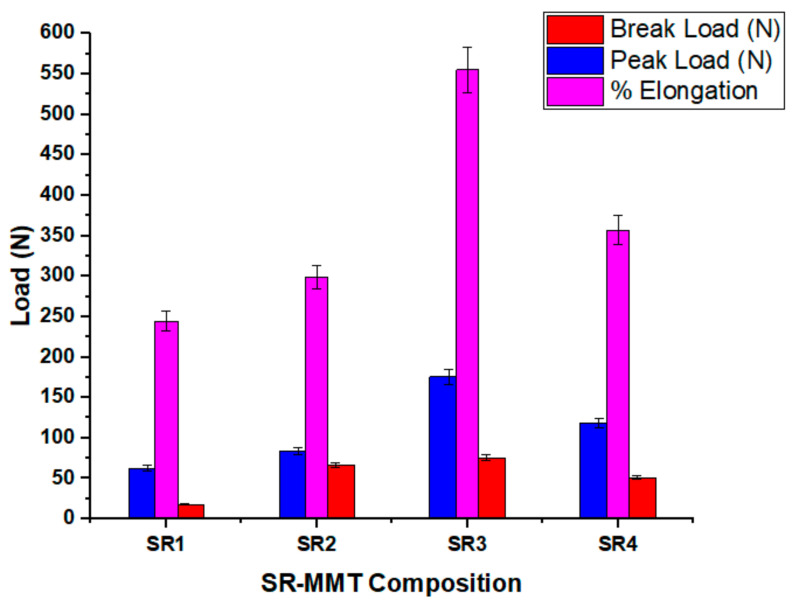
Variation of peak load, break load and % elongation at break for SR–MMT composition.

Figure 6 shows the effect of the concentration of the MMT on the peak load and breaking load. It is observed that the load-carrying capacity of the SR3 composition is the highest amongst all of the compositions. The percentage increase in the peak load is 179.71% as compared to the neat silicon rubber (SR1), and a similar increase is observed in the breaking load. However, with a further increase in the concentration, a reduction in the peak load and a breaking load of 32.4% is observed. These trends can be attributed to the microstructure and interfacial bonding between the SR and MMT.

#### 3.2.4. Percentage Elongation at Break

Figure 6 shows the percentage elongation at break of the SR–MMT nanocomposites for different compositions. The percentage elongation at break increases with an increase in the concentration of the MMT up to a certain percentage. The SR3 composition shows a 56% higher elongation at break as compared with the SR1 composition. Better interlayer bonding for the SR3 composition resulted in the increased percentage elongation. However, a reduction of 35.6% is observed with a further increase in the MMT concentration, which can be attributed to the agglomeration of the MMT particles in the matrix. When the loading of the MMT is more than 5%, clay layers will be less compatible with the SR resulting in the development of a siloxane network [21].

The tensile strength shows a correlation with elongation, i.e., a higher strength for a smaller elongation. The development of a plausible sulfide bond between the backbone chain of the SR and MMT clay improves the degree of cross-linking [22].

#### 3.2.5. Shore D Hardness

**Figure 7:**
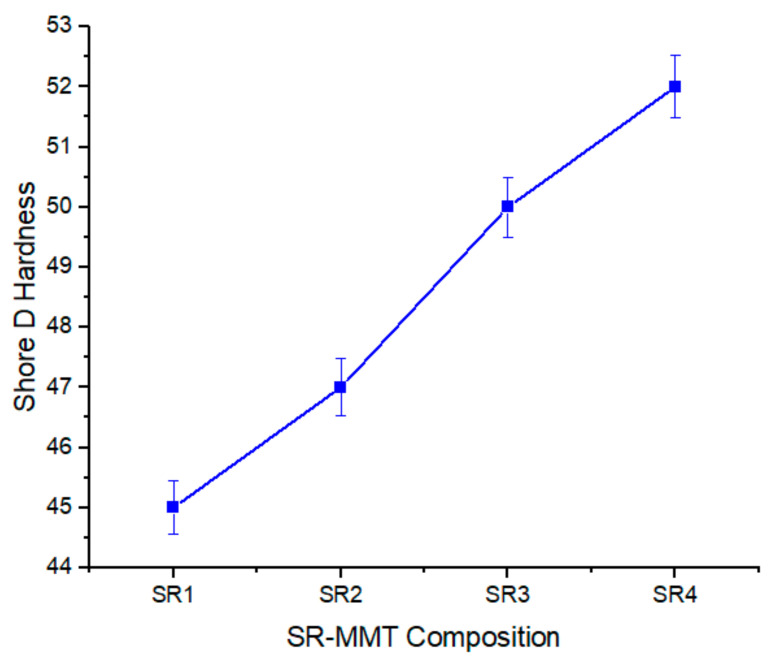
Variation of Shore D hardness for SR/MMT compositions.

The relation of hardness with a concentration of the MMT in the SR/MMT nanocomposites is shown in Figure 7. The amount of MMT tends to increase the hardness of the material. The highest shore D hardness of 52 is observed for SR 4 composition. This increase is almost 16% higher than the SR1 composition. The hardness of the SR can be adjusted by changing the amount of the MMT up to a certain concentration of the MMT. Beyond the SR3 composition, the graph becomes flatter, i.e., the rate at which the hardness increases gets reduced after a certain concentration of the MMT. The increased concentration leads to an agglomeration of the filler causing it to affect not only the mechanical properties, but the mixing process also. Therefore, the optimized amount of the MMT concentration in the SR is 2% as compared with other configurations under study [8].

#### 3.2.6. Tensile Fracture Surface Morphology

**Figure 8:**
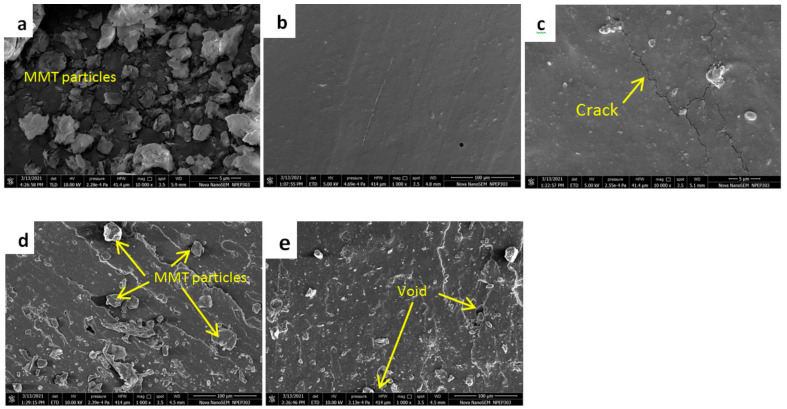
Fracture Surface Morphology for (**a**) MMT powder (**b**) SR1 (**c**) SR2 (**d**) SR3, and (**e**) SR4 composition.

The surface morphology of the fractured surface is significant for getting an insight into the interfacial adhesion between the filler and matrix. Figure 8 shows the fracture surface morphology of all four compositions as well as the MMT powder. Figure 8a shows an SEM image of the MMT powder where the MMT particles are visible. Figure 8b shows the smooth surface for pure silicon rubber, i.e., the SR1 composition. The SR2 composition shows some cracks after tensile loading. These cracks may be due to inefficient interfacial bonding and surface roughness which act as crack initiation points. The increased MMT content is visible from Figure 8d,e. It is exciting to note that the SR3 composition improves the properties due to increased MMT content and smoother distribution of the MMT particles. A further increase in the percentage of MMT increases the number of voids, resulting in a decrease in the mechanical properties for the SR4 composition. Such reduction confirms the start of agglomeration with the increase in the MMT percentage, leading to the generation of voids.

The interfacial adhesion between the silicon rubber elastomer and MMT nanoclay is responsible for enhancing the tensile and tear properties of the nanocomposites. The uniformly distributed MMT in the silicon rubber matrix creates stress concentration points which absorb energy and help to distribute the load uniformly throughout the matrix as in the case of SR3 composition. Further, it acts as a crack arrester under external loading [12].

### 3.3. Tribological Analysis

#### 3.3.1. ANOVA Analysis of Friction Coefficient 

ANOVA test results for the COF are listed in Table 5 below. R-Sq (adj) is 76.60% which represents the significance of the model obtained from experimental observations. The parameter with the highest effect rate load (55.24%) and lowest effect rate is sliding speed (5.65%). Unaccounted factors in the DOE can be counted as errors, at 11.82%. When the load increases to 40 N, the COF is reduced by 20% and the lowest COF is for the SR3 composition; after that, the COF increases. So, the loading of the specimen significantly affects the friction coefficient of the material. For SR3 composition, at 10 N and 30 N load, the COF is 0.710 and 0.340, respectively, which shows a 52.11% reduction.

#### 3.3.2. ANOVA Analysis of Weight Loss

ANOVA test results for weight loss are listed in Table 6 below. The R-Sq (adj) value is 84.12%, representing the significance of the model obtained from experimental observations. The highest effect rate for the filler loading is 55.19%, which significantly influences the weight loss of the material. The theory of plasticity can explain the increase in the weight loss due to an increase in the filler loading. As the content of the filler increases, the stress level also increases, which results in weight. The second significant influencing parameter is the sliding distance which has an effective rate of 27.55%. Unaccounted factors in the DOE can be counted as errors, at 5.43%.

#### 3.3.3. ANOVA Analysis of Specific Wear Rate

ANOVA test results for specific wear rate are listed in Table 7. R-Sq (adj) value is 71.43%, representing the significance of the model obtained from experimental observations. The wear rate is influenced more by the sliding distance with an effective rate of 51.08%. The second largest influencing parameter is the filler content, with a rate effect of 34.95%. The specific wear rate is found to reduce with an increase in the sliding distance. The specific wear rates of SR1 at different sliding distance levels, viz., 1, 2, 3, and 4, are 2.58 × 10^−7^ cm^3^/N-m, 2.37 × 10^−7^ cm^3^/N-m, 1.86 × 10^−7^ cm^3^/N-m, and 8.93 × 10^−8^ cm^3^/N-m, respectively. With an increase in the MMT concentration, the wear rate is reduced drastically from 41% to 60%, corresponding to different sliding distance levels for the SR3 composition. Initially, when the rubber surface is in contact with the steel, heat is generated at a higher rate. This exposes the MMT nanofiller to sliding, resulting in a thin film (transfer film) formation on the contact surface and a reducing of the wear rate [23].

#### 3.3.4. Influence of Normal Load and Sliding Velocity

**Figure 9:**
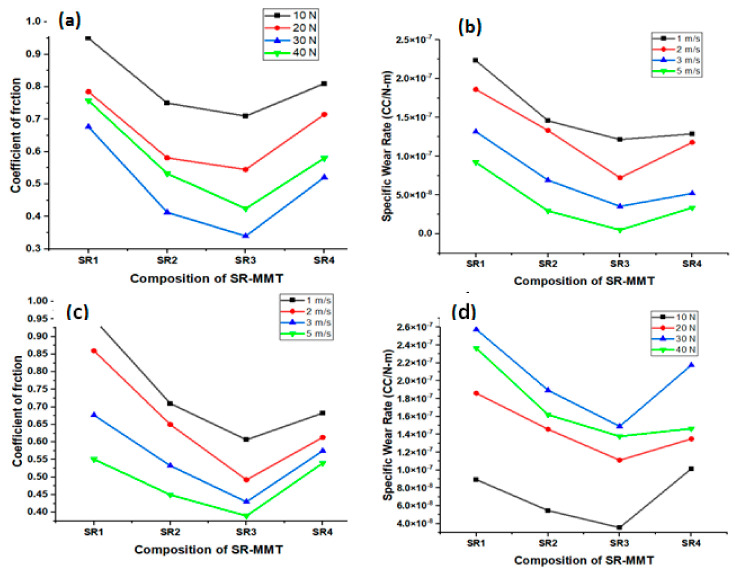
Variation of (**a**) Coefficient of friction (COF), (**b**) Specific wear rate for all compositions at different loads, and (**c**) Coefficient of friction (COF) (**d**) Specific wear rate for all compositions at a different sliding velocity.

Changes in the COF and specific wear rate by changing the load applied and sliding velocity are shown in Figure 9a–d. At 10 N loading, the COF reduces by almost 25%, from 0.950 to 0.710, for the SR3 composition compared with the SR1 composition. At the same time, a 60% reduction of the wear rate is observed at 10 N loading for the SR3 composition as compared to the SR1 composition. The COF and wear rate are the highest for a load of 10 N. Interfacial adhesion is so strong that lower loading cannot break the bond between the constituent elements. Due to this, the material does not break into chips and friction between the surfaces increases. However, for 40 N loading, a dramatic behaviour is observed where the COF reduces, and the specific wear rate increases against the trend due to an increase in the MMT concentration.

With an increase in the MMT content and the sliding velocity, the COF and specific wear rates decrease. For the SR3 composition, the lowest COF and wear rate is observed. When the applied load increases to 30N, a significant reduction in the COF by 52.11% is observed in the SR3 composition. After 30 N the COF is found to increase by 20% to 0.425. When the sliding velocity is lower, more surface-to-surface contact with the friction plate is experienced, resulting in an increasing COF and wear rate. The lubricant film formed between the contacting surfaces is due to the wearing down of polymeric material [24,25]. The transfer layer prevents direct contact between the rubbing surfaces. Generation of the transfer film is another reason for the reduction in the COF as the concentration of the MMT increases. Also, it is evident from the experimental investigation that the SR3 composite shows superior tribological performance for the COF and wear compared to other compositions.

#### 3.3.5. Worn Surface Morphology under Dry Sliding

**Figure 10:**
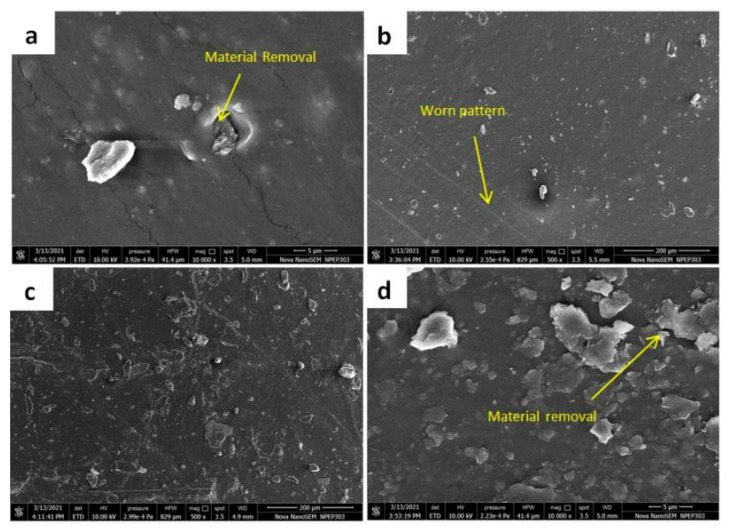
Worn Surface Morphology for (**a**) SR1 (**b**) SR2 (**c**) SR3 and (**d**) SR4 composition after wear test for a load of 40 N and sliding speed of 5 m/s.

SEM images shown in Figure 10 of worn surfaces throw more light on the wear behaviour of the SR and nanocomposites under pin-on-disc wear testing under dry sliding. The rubber material is subjected to two types of friction mechanisms; one is hysteresis, and the other is adhesive friction [26,27]. The rubber specimen tends to deform, causing energy loss due to hysteresis friction.

The surface of the sample and disc advances the adhesive friction. The images are taken for samples and tested at a sliding velocity of 5 m/s and normal loading of 40 N. Pure silicon rubber (SR1) has poor mechanical strength. Hence, it is susceptible to adhesive friction, as shown in Figure 10a. For the SR4 composition, the MMT particles are found to chip off. The particles getting chipped off from the surface form a lubricating film (transfer film) between the disc and specimen surface. The transfer film decreases the adhesive wear, as seen in the SR1 composition. For the SR3 composition, the MMT particles are responsible for reducing the friction due to a reduction in the contact area. Poor bonding between the filler and the matrix leads to chipping of the filler from the matrix which reduces the adhesive friction component. For 5 wt % of the MMT, localized material removal is seen in the SEM imaging.

Adding the MMT beyond the optimum (2 wt %) affects the lubricating film formation due to MMT agglomeration. This agglomeration leads to localized adhesive junctions, increasing both types of friction mechanisms [12]. From the SEM analysis it can be understood that adhesive wear is the leading mechanism of wear. The rubber deforms and glues under the applied load under dry sliding conditions. These glued junctions chip off due to the sliding action. Therefore, the friction initiated by forming the glued junctions is sheared off leading to the removal of the MMT particles and lubricant film formation, resulting in adhesive wear [28].

#### 3.3.6. EDS Spectrum of the Worn Surface

**Figure 11:**
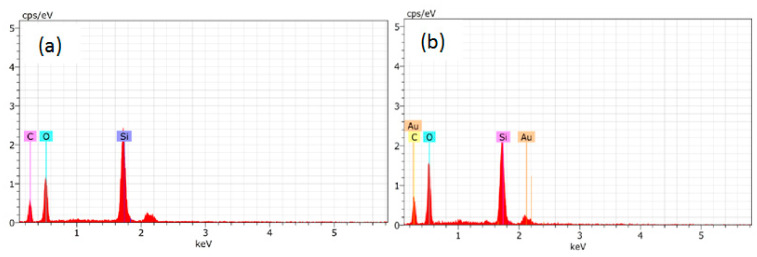
EDS Spectra for (**a**) SR1 and (**b**) SR3 composition after wear test.

Chemical elements on the worn surface can be seen in the EDS spectrums shown in Figure 11. Silicium, carbon, and oxygen are detected for the worn surface under dry sliding conditions. Also, the weight percent of O and C elements found in the SR1 composition are 17.33 and 15.07, respectively, whereas for the SR3 composition they are 15.21 and 17.71. The increased count of the O element and the reduced count of the C element for the SR1 composition indicates more thermal depolymerization than the SR3 composition. Similar elements are available in the pure silicon rubber, which leads to the conclusion that there is no chemical reaction that happened due to the wearing of the specimens.

## 4. Conclusions

A new silicon rubber/MMT nanocomposite was efficaciously fabricated. When explored for mechanical and tribological performance, the MMT–SR nanocomposite exhibited outstanding properties that suit varied applications in the automotive and other fields. Besides, vital tribological parameters like load, sliding speed, and sliding distance were studied using ANOVA. FESEM and EDS analysis of the fractured and worn surfaces was also performed. Following are a few key observations to summarize:

- The SR–MMT nanocomposite with 2% exhibited the highest tensile and tear strength. Compared to SR1, the SR3 composition showed almost 10% and 6% improvement in tensile and tear strength.
- Hardness showed continuous improvement with an increase in the MMT percentage.
- The tribological experiments and its ANOVA revealed that the load has more influence on the friction coefficient while the sliding velocity influences the wear rate.
- A reducing trend of the specific wear rate and the COF was found up to the SR3 composition, and after that, it increased for the SR4 composition. Also, with an increase in the loading, the COF increased but the wear rate decreased. The increasing trend is observed for both the COF and the wear rate, increasing the sliding velocity.
- The surface morphology of different configurations obtained by FE-SEM justifies the results obtained from the mechanical and tribological experiments. The smoother and uniform distribution of the MMT can be observed for the SR3 composition.
- The adhesive wear mechanism is the leading mechanism of wear observed for the developed nanocomposite.

The above conclusion favors the developed nanocomposite from the fundamental analysis and can be confidently used in the automotive field and other suitable applications.

## Figures and Tables

**Table 1:** Properties of Material.

Type/Grade	Silicon Rubber (SH5060 U)	MMT
Specific Gravity (g/cc)	1.15	1.98
Tensile Strength, Ultimate (MPa)	5	101
Elongation (%)	500	8
Hardness, Shore	50 (A)	83 (D)
Particle size (nm)	-	100

**Table 2:** Nanocomposite filler formulations (in weight percent, remaining is SR).

Type/Grade	SR1	SR2	SR3	SR4
MMT	0	0.5	2	5
DCP	2	2	2	2

**Table 3:** Selected levels of different parameters.

Parameter	Level			
Composition	SR1	SR2	SR3	SR4
Load (N)	10	20	30	40
Sliding Speed (m/s)	1	2	3	4
Sliding Distance (mm)	1000	1800	2600	3200

**Table 4:** Taguchi L_16_orthogonal array for the design of tribological experiments.

Exp. No.	Composition	Load	Sliding Speed	Sliding Distance
1	SR1	1	1	1
2	SR1	2	2	2
3	SR1	3	3	3
4	SR1	4	4	4
5	SR2	1	2	3
6	SR2	2	1	4
7	SR2	3	4	1
8	SR2	4	3	2
9	SR3	1	3	4
10	SR3	2	4	3
11	SR3	3	1	2
12	SR3	4	2	1
13	SR4	1	4	2
14	SR4	2	3	1
15	SR4	3	2	4
16	SR4	4	1	3

**Table 5:** ANOVA for Coefficient of friction.

Source	DF	Sum of Squares (SS)	Mean Square (MS)	F-Value	*p*-Value	Effect Rate (%)
Composition (%)	1	0.08102	0.08102	2	0.185	6.59
Load (N)	1	0.67881	0.37881	9.36	0.011	55.24
Sliding Speed (m/s)	1	0.06949	0.06949	1.72	0.217	5.65
Sliding Distance (mm)	1	0.25437	0.25437	6.28	0.029	20.70
Error	11	0.14525	0.04048			11.82
Total	15	1.22894				100.00
Significance	R-sq = 80.95%			R-sq (adj) = 76.60%		

**Table 6:** ANOVA for Weight Loss.

Source	DF	Sum of Squares (SS)	Mean Square (MS)	F-Value	*p*-Value	Effect Rate (%)
Composition (%)	1	0.000434	0.000434	1.64	0.226	8.11
Load (N)	1	0.002953	0.000343	1.3	0.278	55.19
Sliding Speed (m/s)	1	0.000199	0.000199	0.75	0.404	3.72
Sliding Distance (mm)	1	0.001474	0.001474	5.58	0.038	27.55
Error	11	0.000291	0.000264			5.43
Total	15	0.005351				100.00
Significance	R-sq = 92.22 %			R-sq (adj) = 84.12 %		

**Table 7:** ANOVA for specific wear rate.

Source	DF	Sum of Squares (SS)	Mean Square (MS)	F-Value	*p*-Value	Effect Rate (%)
Composition (%)	1	7 × 10^−6^	0.000007	1.54	0.241	7.53
Load (N)	1	3.25 × 10^−5^	0.00001	2.09	0.176	34.95
Sliding Speed (m/s)	1	1 × 10^−6^	0.000001	0.19	0.67	1.08
Sliding Distance (mm)	1	4.75 × 10^−5^	0.000025	5.37	0.041	51.08
Error	11	5 × 10^−6^	0.000005			5.38
Total	15	93 × 10^−6^				100.00
Significance	R-sq = 83.04%			R-sq (adj) = 71.43%		

## Data Availability

The data presented in this study are available on request from the corresponding author.
